# A double-layer PLGA/CoI-MeHA tissue engineering scaffold for urethral reconstruction

**DOI:** 10.3389/fphar.2025.1555183

**Published:** 2025-02-17

**Authors:** Mingyang Chang, Qinyuan Tan, Ge Bian, Ming Zhang, Jianing Lv, Junjie Su, Xiaoqing Wang

**Affiliations:** ^1^ Department of Urinary Surgery, The First Hospital of Jilin University, Jilin University, Changchun, China; ^2^ Department of Urology, The People's Hospital of Jimo, Qingdao, China

**Keywords:** urethra, urethral injury, scaffold, reconstruction, tissue engineering

## Abstract

**Introduction:**

Urethral injury caused by various reasons usually leads to urethral stricture. And severe urethral stricture can further induce complications such as bladder stones, fistulas, sepsis, and even renal failure. At present, surgical methods such as urethral reconstruction and end-to-end anastomosis are commonly used to solve this problem. But this treatment method often has a high recurrence rate. So simply relying on the repair of surrounding autologous tissue cells to reconstruct the urethra is difficult to achieve long-term stability, and constructing a suitable urethral graft is an effective and feasible solution.

**Methods:**

Here, we designed and prepared a double-layer PLGA/CoI-MeHA tissue engineering scaffold to better simulate the natural anatomy of the urethra and achieve urethral tissue regeneration and reconstruction in patients with urethral stricture and Hypospadias caused by various reasons. The double-layer tissue engineering scaffold was generated using electrospinning and light curing technology.

**Results:**

Through electrospinning and light curing technology, we successfully screened the PLGA/CoI (7:3) electrospun membrane and MeHA (40.72%) hydrogel. Furthermore, we successfully prepared PLGA/CoI-MeHA bilayer urethral stents loaded with rabbit urethral smooth muscle cells and rabbit urethral epithelial cells, respectively, and achieved favorable results for urethral defect repair and urethral reconstruction in rabbits. The mechanical characterization of the scaffold indicates that it has sufficient mechanical strength to meet experimental and clinical needs. In addition, it showed satisfactory biocompatibility in cell experiments and in the in vitro degradation experiments. The double-layer urethral stents demonstrated exceptional performance in repairing urethral defects in rabbits.

**Discussion:**

We had successfully designed and prepared a double-layer PLGA/CoI-MeHA tissue engineering scaffold. The stent displayed sufficient mechanical strength, good biocompatibility and degradation characteristics, and effectively simulated the natural anatomy of urethra, achieving satisfactory urethral defect reconstruction results.

## 1 Introduction

Factors leading to urethral injury include malformations, inflammation, and trauma, which can often lead to urethral stricture, resulting in bladder stones, fistula, sepsis, and kidney failure (Lumen et al., 2021; Wessells et al., 2023). At present, different repair strategies based on the length, location, and causes of urethral injury are used (Marshall et al., 2015; Casarin et al., 2022). Until now, urethral injuries involving the corpora cavernosa tissue can be cured by surgical intervention. And the commonly used surgical procedures include urethroplasty and end-to-end anastomosis. However, the recurrence rate is high (Wang et al., 2024; Sedigh et al., 2023). Long term urethral injury greatly reduces the repair potential of surrounding tissues. The proliferation and migration of self cells make it difficult to achieve urethral reconstruction (Cheng et al., 2018). Thus, it is imperative to fabricate fitting urethral grafts to achieve efficient urethral restoration (Li et al., 2024).

Numerous medical procedures for treating urethral injuries involve the use of the patient’s own tissue, such as skin flaps or tissue from the inside of the cheek (Chapple et al., 2014; Tan et al., 2022; Fuehner et al., 2021; Barratt et al., 2021; Sánchez et al., 2023a; Sterling et al., 2024). However, flap harvesting and transplantation is a technically complex procedure, and this treatment option is not always available (Versteegden et al., 2017). These treatments, however, come with their own set of drawbacks, including damage to the area from which the tissue is taken, the formation of abnormal connections between body parts (fistulas), and a high likelihood of the injury recurring in the area that was fixed (Xu et al., 2015; Kurtzman et al., 2021). The fusion of cells with scaffolds allows the tissue-engineered urethra to emulate the characteristics of a natural urethra (Guo and Ma, 2018; Song et al., 2022; Wang et al., 2022; Hu et al., 2023; Meng et al., 2023). Consequently, urethral scaffolds are designed to be conducive to cell proliferation and provide the necessary mechanical strength. And it acts as a bridge for cell proliferation and migration (Farzamfar et al., 2022). The architecture of the graft is pivotal to the urethral reconstruction process, influencing both the microscopic and macroscopic aspects of the procedure (Huang et al., 2023; Xuan et al., 2022). Properly designed grafts can foster tissue regeneration that more closely resembles the natural urethra, which in turn can expedite the healing process and enhance the likelihood of a successful outcome.

**FIGURE 1:**
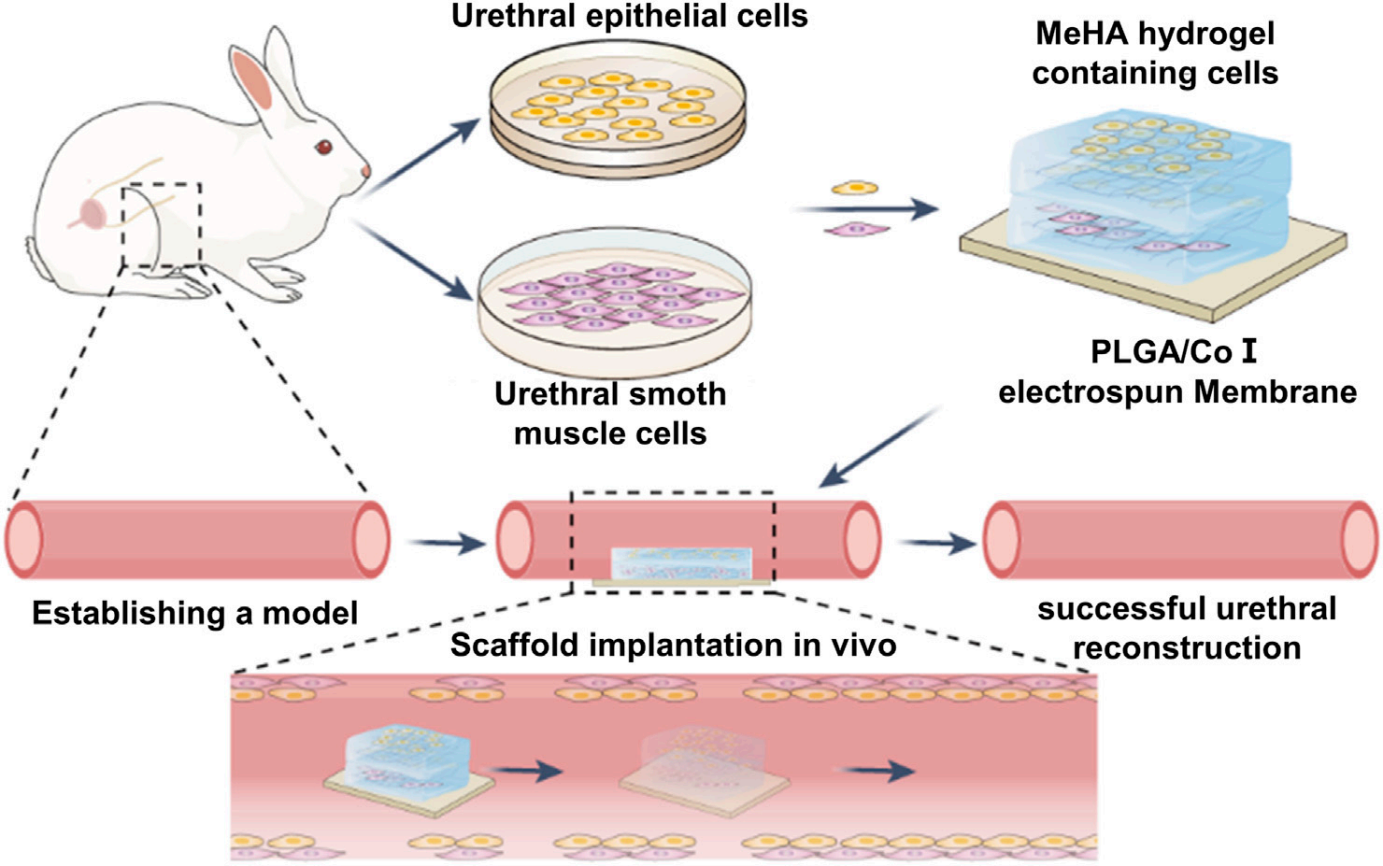
Synthesis of hydrogel scaffolds and *in vivo* repair process.

Therefore, we designed a double-layer polylactic-co-glycolic acid (PLGA)/collagen type I (CoI)-methacrylated hyaluronic acid (MeHA) tissue engineering scaffold, which simulates the urethra’s natural microenvironment through the integration of cells with a supportive scaffold, effectively solving the abovementioned problems (Figure 1). This scaffold serves a dual purpose: it offers essential mechanical support while also fostering an optimal environment conducive to cell growth and proliferation. As a result, the regeneration of urethral tissue tends to approach the natural urethra, and the success rate of repair will be significantly improved.

## 2 Materials and methods

### 2.1 Materials

PLGA was purchased from Changchun Shengboma Biomaterials Co., Ltd. Hyaluronic acid (HA) was purchased from Bloomage Freda Co., Ltd. Methacrylic anhydride (MA) was purchased from Shanghai Macklin Biochemical Technology Co., Ltd. Hexafluoroisopropanol (HFIP) was purchased from Shanghai Yuanye Biotechnology Co., Ltd. Phenyl-2,4,6-trimethylbenzoylphosphite was purchased from Shanghai Yuanye Biotechnology Co., Ltd. Lithium acid (LAP) and hyaluronidase (HAase) were purchased from Shanghai Yuanye Biotechnology Co., Ltd. Sodium hydroxide was purchased from Shanghai Lingfeng Chemical Reagent Co., Ltd. Collagen type I (CoI) was purchased from Beijing Suo Laibao Technology Co., Ltd. Paraformaldehyde was purchased from Beijing Aobosen Biotechnology Co., Ltd. Aetna was purchased from Xiamen Demeco Biotechnology Co., Ltd. Absolute ethanol was purchased from Sinopharm Group Pharmaceutical Co., Ltd. Gentamicin sulfate injection was purchased from Hebei Rongrun Biotechnology Co., Ltd. Compound diatrizoate meglumine injection was purchased from Xi’an Hanfeng Pharmaceutical Co., Ltd. The complete culture medium of rabbit urethral smooth muscle cells was purchased from Wuhan Punuosai Life Technology Co., Ltd. The complete culture medium of rabbit urethral epithelial cells was purchased from Beijing Beina Chuanglian Biotechnology Research Institute. Trypsin was purchased from Sigma United States. Phosphate buffered saline (PBS) was purchased from Boster China. The CCK-8 kit was purchased from Shanghai Biyuntian Biotechnology Co., Ltd. The live cells/dead cells staining kit was purchased from Shanghai Beibo Biotechnology Company.

### 2.2 Preparation of the PLGA/CoI electrospun membrane

The PLGA particles (molecular weight: 100,000 g/mol) and CoI (mass ratio of the two: 7:3/8:2) were dissolved in HFIP to a solution concentration of 15%w/v. Under normal temperature conditions, the mixed solution was stirred for 8 h on a magnetic stirrer to achieve a stable spinning solution. Then it was aspirated into a 1 mL syringe, and a 19-G copper blunt needle was used for electrospinning. The spinning parameters were as follows: spinning voltage 25 kV, injection speed 1 mL/h, and copper foil receiving distance 15 cm. The PLGA/CoI electrospun membrane obtained after spinning for 5 h was subjected to vacuum drying for 24 h to remove non-volatile solvents, and then transferred to a conventional drying oven for later use.

### 2.3 Surface morphology of the PLGA/CoI electrospun membrane

The PLGA/CoI electrospun membrane was cut into a 5 × 5 mm sheet. The membrane was stuck to a conductive adhesive with the copper foil receiving surface as the upper surface, and the surface of the Gold 30 s electrospun membrane was sprayed. The surface morphology of the electrospun membrane was examined using Zeiss73447 scanning electron microscope operated.

### 2.4 Density and porosity test of the PLGA/CoI electrospun membrane

The PLGA/CoI electrospun membrane was cut into sheets of a similar size. The initial mass of a single electrospun membrane was defined as M. The membrane was immersed in ethanol of a known volume (V1) and soaked for 5 min. At this point, the total volume of ethanol and the electrospun membrane was defined as V2. The electrospun membrane was removed, and the volume of the residual ethanol was defined as V3. Subsequently, the total volume of the electrospun membrane was calculated as V = V2 – V1. The density and porosity of the electrospun membranes were calculated by the following formulas:

Density (g/cm^3^)

$$\rho =\frac{M}{V2-V3}$$

Porosity (%)

$$\varepsilon =\frac{V1-V3}{V2-V3}\times 100\%$$

### 2.5 Tensile mechanical properties test of the PLGA/CoI electrospun membrane

The PLGA/CoI electrospun membrane was cut into 4 × 1 cm thin slices. The property of electrospun membrane was tested using a mechanical experiment analyzer equipped with a 50 N mechanical sensor. A stretching jig was used to clamp 1 cm above and below the sheet, which was stretched at a loading rate of 0.5 mm/min until the sheet was torn.

### 2.6 *In vitro* degradation performance of the PLGA/CoI electrospun membrane

Artificial urine and PBS solution were used for *in vitro* degradation. The PLGA/CoI electrospun membrane was dried in a vacuum drying oven and weighed, followed by immersion in urine and PBS, respectively, before placing in a constant temperature oscillator adjusted to 37°C. The liquid was replaced every 3 days. Three parallel samples were obtained at the 1st, 2nd, 4th, 6th, 8th, and 10th week. The residual weight rate was measured after observing the general shape as follows:

Residual weight rate (%)

$$\text{Residual weight rate}=\frac{\text{residual}\;\text{weight}}{\text{first}\;\text{weight}}\times 100\%$$

### 2.7 Biocompatibility of the PLGA/CoI electrospun membrane

The electrospun membrane was cut into several thin slices, 1.5 × 1.5 cm in size, and sterilized overnight in an ozone ultraviolet disinfection cabinet to ensure that they are fully sterilized. Three thin slices were placed in a 24-well plate, fixed with polytetrafluoroethylene gaskets that had been sterilized in ethanol overnight, and 1 mL of complete medium with 50,000 rabbit urothelial cells/urethral smooth muscle cells were added into each well. Within the control group, complete medium and cells were directly added to the well plate. After 24, 48, and 120 h, the cells were treated with the CCK-8 mixture (medium: CCK-8 = 9:1) for 2 h. And measuring the absorbance to calculate the biocompatibility of the electrospun membrane. Three parallel samples were tested in each group.

### 2.8 Preparation of MeHA

A total of 500 mg of HA (molecular weight: 1.5 million g/mol) was added to 100 mL of deionized water and then stirred for 12 h. Three samples were prepared. Under ice-bath conditions, 1.5, 2, or 2.5 mL of MA was added to the solution. The reaction was continued under ice-bath conditions for 24 h. Then it was dialyzed in deionized water for 3 days with the water changed every 8 h. Subsequently, the dialyzed solution was dehydrated using a freeze dryer to obtain a white flocculent product.

### 2.9 MeHA hydrogel compression mechanical performance test

The universal mechanical experiment analyzer equipped with a 50 N mechanical sensor was used to perform the compression test. The MeHA hydrogel pre-polymerization liquid was placed in the polytetrafluoroethylene mold and combined into a cuboid shape. Following blue light irradiation for 10 s, a gel sample was obtained. Compression experiments were performed at a loading rate of 5 mm/min.

### 2.10 MeHA hydrogel rheological properties

The MeHA hydrogel pre-polymerization solution was placed in a quartz mold. And after blue light irradiation, the acquired gel specimen was positioned within the rheometer’s fixture. Set the test parameters to frequency (1 Hz) and strain (0.5%).

### 2.11 *In vitro* degradation performance of the MeHA hydrogel

A total of 300 μL of hydrogel pre-polymerization solution was added to a quartz bottle, and blue light irradiation for 10 s was used to obtain a gel sample. The sampled were placed in in 1 mL of PBS (without enzyme) and PBS solution containing 0.075 mg/mL of hyaluronidase (HAase). Continuous shaking at 37°C was performed in a constant temperature shaking box. The next day, the quartz bottle containing the hydrogel sample was removed, the upper solution was carefully absorbed on the surface of the hydrogel with absorbent paper. Once the residual water was removed, the gel was weighed, and the percentage of the gel weight of the original weight at every time point was calculated.

### 2.12 MeHA hydrogel biocompatibility

MeHA was sterilized overnight using an ozone ultraviolet (UV) sterilizer. MeHA was then dissolved at a concentration of 1% w/v using complete medium. Rabbit urothelial cells/urethral smooth muscle cells were planted in a 96-well plate. Each well was supplemented with 200 μL of complete medium and then incubated for a period of 24 h. The MeHA solution was serially diluted seven times to obtain eight concentration gradients. The upper medium was aspirated, and 200 μL of each MeHA solution with different concentration gradients were sequentially added to the wells. Complete medium was added to the control group. The cells were subjected to the CCK-8 mixture at intervals of 24, 72, and 120 h. And measuring the absorbance to calculate the biocompatibility of the MeHA. Each group tested five parallel samples. Subsequently, the cells were digested and counted with trypsin. And then added to the hydrogel pre-polymerization solution, vortexed for 30 s, mixed evenly, and added dropwise to a 24-well plate. The polymerization solution was irradiated with blue light for 10 s to allow it to solidify, and 1 mL of complete medium was added to submerge the cells. The plates were incubated in a cell culture chamber overnight. The next day, the culture medium was aspirated off the upper layer of the gel, the live/dead staining solution diluted with dPBS was added to every well. The viability and mortality of the cells were assessed using a fluorescence microscope.

### 2.13 Extraction of rabbit urothelial cells and rabbit urethral smooth muscle cells

Male New Zealand white rabbits were anesthetized using acetylene and kept under anesthesia using 2% isoflurane. An incision was performed superior to the pubic symphysis to reveal the bladder. A biopsy specimen measuring 2 by 2 cm was excised from the bladder wall. The sample was rinsed with PBS solution containing antibiotics. It was subsequently incubated overnight at 4°C with the application of Dispase type 2 enzyme. The inner layer’s epithelium was removed, then the tissue was sectioned into smaller fragments and soaked in a 0.25% trypsin solution for a duration ranging from 15 to 30 min. The final solution with bladder epithelial cells was cultured rabbit urothelial cell complete medium.

Fresh bladders were obtained from male New Zealand White rabbits after euthanasia with chloral hydrate using a sterile scalpel. The muscular part of the tissue was subsequently dissected under microscopic guidance. It was finely chopped and then treated with a 0.5% (weight/volume) solution of Type I collagenase for a period of 30 min. The isolated cells were placed in a 10 cm tissue culture dish with rabbit urethral smooth muscle cell complete medium (Pernosai, Wuhan, China).

### 2.14 Identification of rabbit urothelial cells and rabbit urethral smooth muscle cells

Immunofluorescence identification of rabbit urothelial cells was performed using cytokeratin 19 (CK-19). After the cells climbed onto the slices, the culture medium was aspirated, fixed with 4% PFA. And washed three times for 5 min with PBS. The slides were dehydrated and placed on petri dish supports. TritionX-100 (0.5%) was mixed with PBS at a 1:1 ratio, and 10% serum was added form the blocking solution. Next, 50 μL of membrane rupture sealing solution was dropped onto the waterproof membrane, and the side of the slide with cells was covered for 2 h. Anti-CK-19 was diluted with PBS (1:100). And 50 μL was added to the waterproof membrane (in a wet box), followed by incubation at 4°C. Next, cells were incubated with secondary antibody in the dark for 2 h, followed by washing three times with PBS. The cells were then stained with DAPI. Subsequently, the samples were rinsed with PBS for a total of three times, with each rinse lasting for 5 min. One drop of Fluoromount-G was added to each slide, which was covered and the imaged using a fluorescent inverted microscope.

Immunofluorescence identification of rabbit urethral smooth muscle cells was performed using alpha smooth muscle actin (α-SMA). The slides with cell adhesion on them underwent a series of washing steps with PBS, three times for 3 min each, followed by fixation with 4% PFA. And then they were washed again with PBS, three times for 3 min each. TritionX-100 (0.5%) was mixed with PBS at a 1:1 ratio, which was added to the cells to permeate, followed by washing with PBS. Excess PBS was blotted, then serum was added in a dropwise manner on the plectrum. Cells were blocked for 30 min. After the removal of the blocking solution, an adequate volume of the diluted primary antibody solution, specifically targeting α-SMA, was applied to each slide. On the following day, the cells underwent a washing process with PBS, which was repeated three times. The excess liquid was absorbed. And the fluorescent-labeled secondary antibody was added in a dropwise manner. These cells were incubated in a wet box at 37°C during 1 h. Cells were incubated with DAPI in the dark for a period of 5 min to facilitate the staining of their nuclei. Excess DAPI was washed off with PBS four times for 5 min each time. Excess liquid on the slide was blotted. The slide was covered with a sealing medium. Cells were imaged using a fluorescence microscope.

### 2.15 Preparation of the PLGA/CoI-MeHA urethral scaffold

The PLGA/CoI electrospun membrane was cut into 2 × 1.5 cm thin slices and placed on a box-shaped polytetrafluoroethylene mold. A circular polytetrafluoroethylene mold was used to press and fix the electrospun membrane. Two MeHA pre-polymerization solutions were prepared, and the rabbit urothelial cells and rabbit urethral smooth muscle cells were incorporated into the pre-polymerization mixture at a density of 1 × 108/mL, followed by vortexing for 30 s to evenly distribute the cells. A 100 μL pipette was used to inject 60 μL of the prepolymer solution containing rabbit urethral smooth muscle cells into the hollow part of the mold, which was then irradiated with blue light for 1 s before adding 40 μL of the prepolymer solution containing rabbit urethral epithelial cells, and irradiating with blue light for 10 s. The electrospun membrane’s surface became coated with a gel as the polymer solution solidified. The mold was then removed to obtain the PLGA/CoI-MeHA urethral scaffold.

### 2.16 Rabbit urethral defect model establishment and scaffold implantation

Forty male New Zealand white rabbits were equally divided into four groups: the PLGA/CoI-MeHA urethral scaffold implanted with cells, PLGA/CoI-MeHA urethral scaffold without implanted cells, autologous tissue suture after urethrotomy (gold standard), and no treatment after urethrotomy (blank control). All animals were anesthetized by intramuscular injection of Antai and maintained with 2% isoflurane. The anterior urethra of the rabbits was fully separated, and the ventral urethral defect model was established by resecting the ventral side at 1 cm and 0.5 cm. A 5–0 suture was used to match the scaffold with the defect. A 6Fr catheter was inserted into the urethra in all groups, and free urination was performed 7 days after operation. Gentamicin (0.5 mL) was injected intramuscularly 3 days after the operation. Three months later, retrograde urethrography was performed with contrast agent (meglumine diatrizoate), and the repaired urethra was removed for histopathological characterization.

### 2.17 Statistical analysis

Each test was carried out independently a minimum of three times, with results shown as mean ± standard deviation (*s*). Data analysis was conducted using SPSS (version 13.0, United States); measurement data were compared using the *t*-test, while group differences were assessed through variance analysis. Levels of significance were noted as *p < 0.05, **p < 0.01, ***p < 0.001, and ****p < 0.0001.

## 3 Results

### 3.1 Preparation and characterization of PLGA/CoI electrospun membranes

**FIGURE 2:**
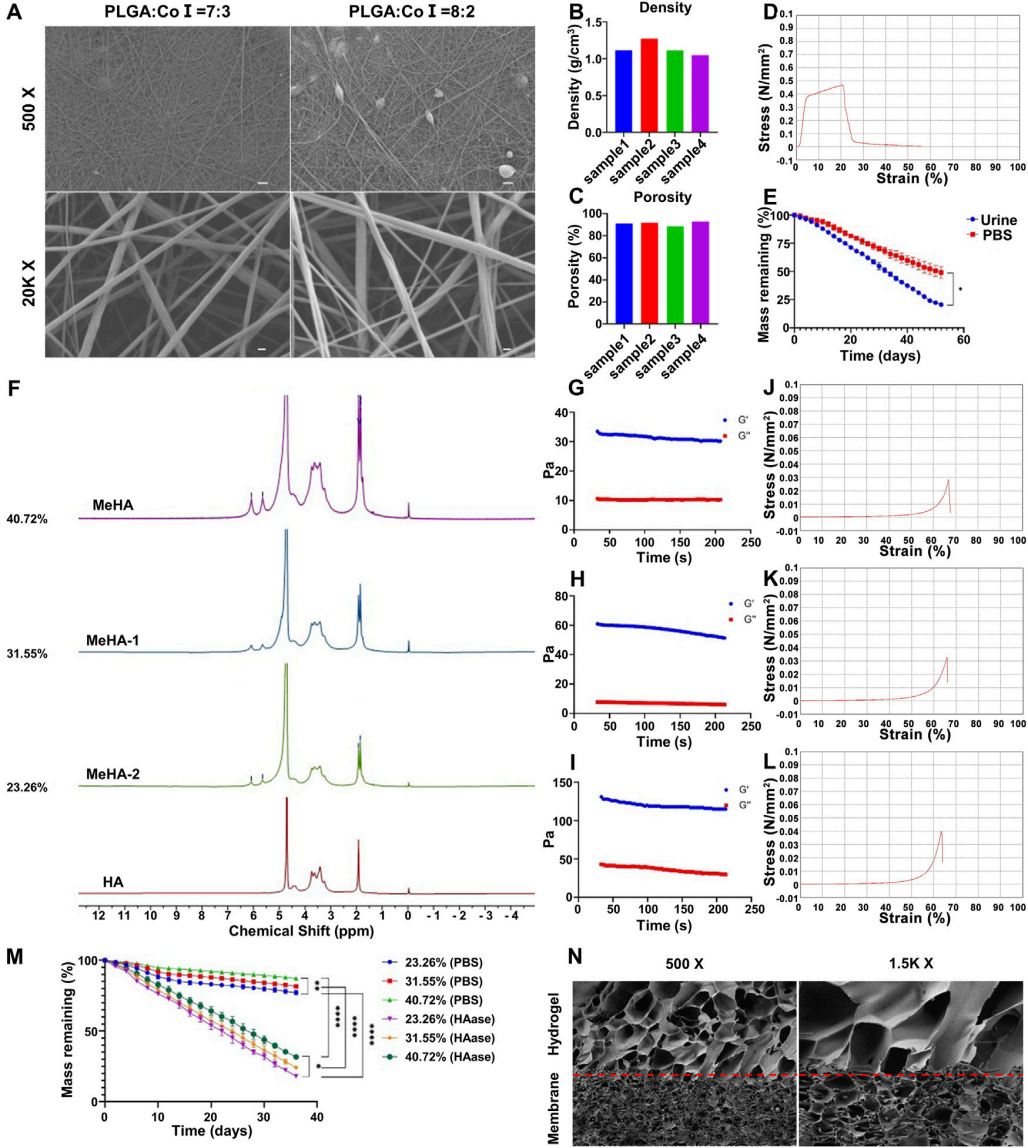
Characterization of the double-layer polylactic-co-glycolic acid (PLGA)/collagen type I (CoI)-methacrylated hyaluronic acid (MeHA) urethral tissue engineering scaffold. **(A)** Morphology of the PLGA/CoI electrospun membranes with different ratios observed by scanning electron microscopy. **(B)** Density of PLGA/CoI (7:3) electrospun film. **(C)** Porosity of the PLGA/CoI (7:3) electrospun membrane. **(D)** Tensile properties of the PLGA/CoI (7:3) electrospun film. **(E)** *In vitro* degradation characteristics of the PLGA/CoI (7:3) electrospun membrane in PBS and artificial urine. **(F)** Proton nuclear magnetic resonance (1H-NMR) with different grafting rates of MeHA hydrogels. **(G–I)** The rheological properties of MeHA hydrogels with grafting rates of 23.26%, 31.55% and 40.72%, respectively. **(J–L)** The compression properties of MeHA hydrogels with grafting rates of 23.26%, 31.55% and 40.72%, respectively, were tested. **(M)** *In vitro* degradation characteristics of the MeHA hydrogels with or without hyaluronidase. **(N)** The morphology of the PLGA/CoI-MeHA urethral tissue engineering scaffold at the membrane-glue interface under scanning electron microscopy.

PLGA and CoI are both materials with good biocompatibility, so this work used electrospinning technology to prepare PLGA/CoI electrospun membranes. Scanning electron microscopy (SEM) showed that the nanofiber diameter of the PLGA/CoI (7:3) electrospun film was 144.13 ± 38.22 nm (mean ± variance), and that of PLGA/CoI (8:2) electrospun film was 129.39 ± 69.71 nm. Compared with PLGA/CoI (8:2) electrospun film, PLGA/CoI (7:3) electrospun film had fewer spindle-shaped lumps, and the diameter and distribution of nanofibers were more uniform and stable (Figure 2A). Therefore, for the subsequent experiments of this research, the PLGA/CoI (7:3) electrospun membrane was chosen.

The density and porosity of the PLGA/CoI (7:3) electrospun membrane were obtained by measurement and calculation. The density of the electrospun membrane was 1.135 ± 0.09 g/cm^3^ and the porosity was 90.99% ± 3.39% (Figures 2B,C). The density of electrospun film was higher than that of water, and the reticular structure of the nanofibers was compact. The high porosity was favorable for the MeHA prepolymer, enabling it to penetrate into the electrospun membrane and firmly combine with the electrospun film after light curing. The mechanical tensile properties test showed that the tensile force of the electrospun film exceeded 0.4 Mpa, which could fully meet the needs of surgical sutures, and provided a mechanical strength basis for subsequent animal experiments (Figure 2D). The degradation rate of the PLGA/CoI (7:3) electrospun membrane in urine was faster than that in PBS. The electrospun membrane had degraded in artificial urine after 52 days, which provides a sufficient urethral repair time (Figure 2E).

### 3.2 Preparation and characterization of MeHA

HA and MeHA were characterized by proton nuclear magnetic resonance (1H-NMR). A comparison of MeHA and HA NMR showed that after the reaction with methacrylic anhydride, HA has two new signal peaks at δ = 6.05 ppm and 5.65 ppm, and a bifurcated peak at δ = 1.8 ppm. These three peaks were the nuclear magnetic peak of double bond hydrogen on the methacrylate group and the nuclear magnetic peak of methyl hydrogen, respectively. The nuclear magnetic peak at δ = 1.9 ppm was the nuclear magnetic peak of methyl hydrogen on the side chain of HA. Furthermore, with the increase in the MA input content, the intensity of the NMR peaks at δ = 6.08 ppm, 5.65 ppm, and 1.8 ppm also increased. When comparing the integral area of methacrylate hydrogen with that of HA methyl hydrogen, the degree of methacrylate esterification of HA can be obtained. Therefore, the grafting rates of MeHA-1, MeHA-2, and MeHA were calculated as 23.26%, 31.55%, and 40.72%, respectively (Figure 2F). To summarize, with the increase in the MA content, the grafting rate of MeHA increased obviously. On the other hand, the degree of double bonding in HA will influence the physical and chemical properties of the subsequent UV-cured hydrogel.

The rheological properties of MeHA hydrogels with different grafting ratios were tested. Once the hydrogel had formed following UV-curing for 10 s, the storage modulus G′ and energy dissipation modulus G″ were tested under the conditions of constant frequency (1 Hz) and constant strain (0.5%). As can be seen from the figure, the hydrogel with a grafting rate of 40.72% has the highest G′, which proves that the increase in the grafting rate can increase the elasticity of the hydrogel. Similarly, with the significant increase in G″, it shows that the increase in the grafting rate may increase the viscosity of the hydrogel to a certain extent (Figures 2G–I).

To meet the needs of subsequent animal experiments, the hydrogel must have a certain mechanical strength to support its structure and cell survival. Therefore, the compression properties of MeHA hydrogels with different grafting ratios were tested. When the content of MeHA was 0.5%, the maximum strain of the MeHA hydrogel with a 23.26% grafting ratio was 66.75%, and the maximum compressive strength was 28.43 kPa (Figure 2J). When the grafting rate was 31.55%, the maximum strain of the MeHA hydrogel was 65.85%, and the maximum compressive strength was 32.76 kPa (Figure 2K). Furthermore, when the grafting rate was 40.72%, the maximum strain of the MeHA hydrogel was 63.73%, and the maximum compressive strength was 38.13 kPa (Figure 2L). Therefore, with the increase in the grafting ratio of MeHA, the maximum compressive strength of the hydrogel gradually increased and the maximum strain gradually decreased.

We performed *in vitro* degradation experiments on the MeHA hydrogels with different grafting ratios, which showed that the degradation rate of hydrogels in PBS solution containing 0.075 mg/ML HAase was significantly faster than that in PBS. Furthermore, the degradation rate of hydrogels with a high grafting rate of MeHA was faster than that of those with a low grafting rate. In the PBS solution containing HAase, the hydrogel was degraded in 36 days, which could fully meet the needs of animal experiments (Figure 2M). To summarize, under the condition of ensuring sufficient mechanical strength, we chose MeHA with a grafting rate of 40.72% for follow-up experiments.

Urethral tissue repair scaffolds were successfully fabricated with selected the PLGA/CoI electrospun membrane and MeHA hydrogel, and their microstructure was characterized by SEM. The composite urethral tissue engineering scaffold was successfully prepared, and the hydrogel prepolymer fully immersed the electrospun membrane to form a stable scaffold structure (Figure 2N).

### 3.3 Immunofluorescence staining of rabbit urethral epithelial cells and rabbit urethral smooth muscle cells

**FIGURE 3:**
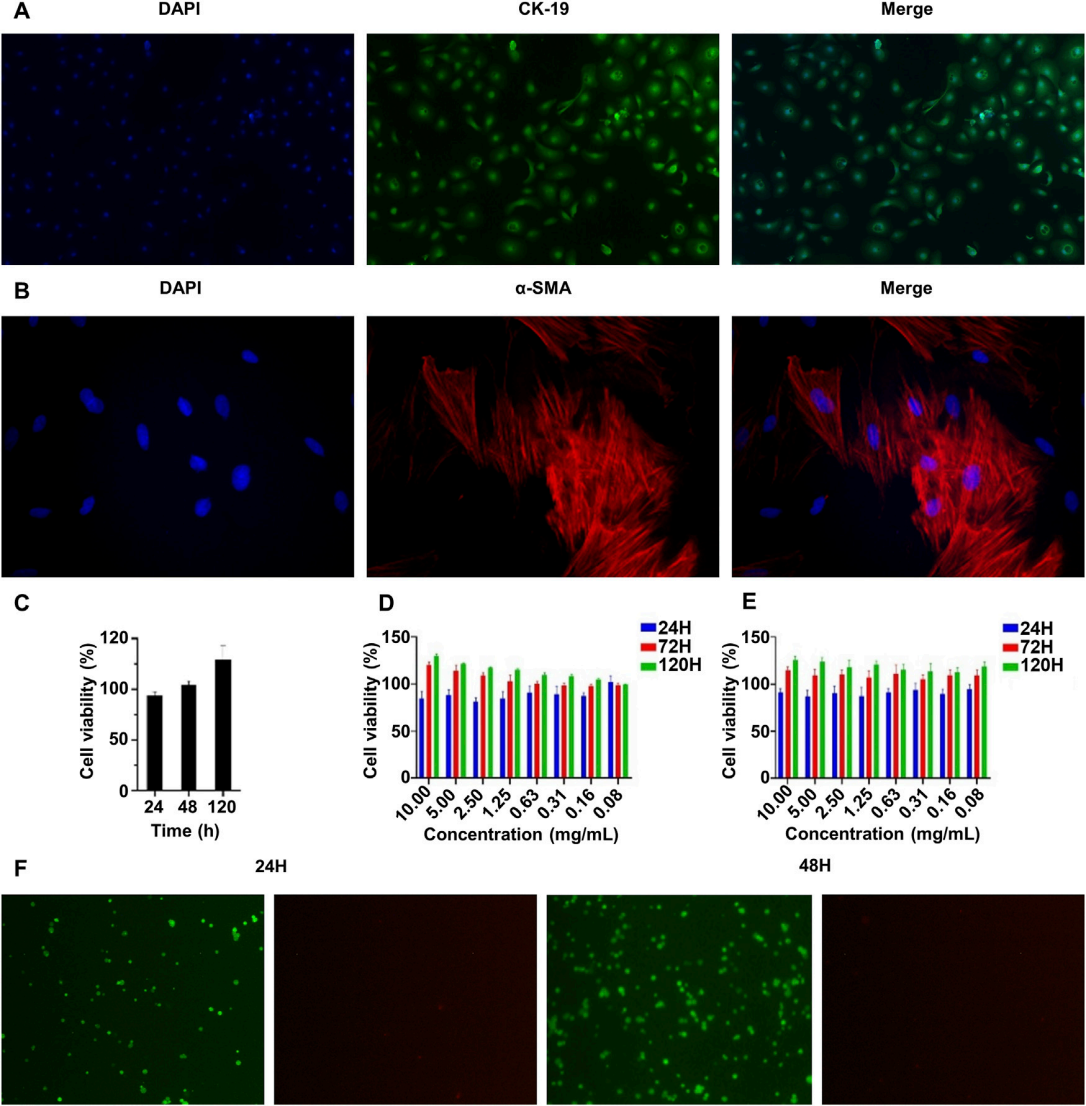
Characterization of the biocompatibility of the double-layer polylactic-co-glycolic acid (PLGA)/collagen type I (CoI)-methacrylated hyaluronic acid (MeHA) urethral tissue engineering scaffolds. **(A)** CK-19 immunofluorescence staining of rabbit urethral epithelial cells. **(B)** α-SMA immunofluorescence staining of rabbit smooth muscle cells. **(C)** The proliferation characteristics of rabbit urethral smooth muscle cells on the PLGA/CoI electrospun membrane were measured by the CCK-8 assay. **(D, E)** The proliferation characteristics of rabbit urethral epithelial cells and smooth muscle cells in the MeHA hydrogel were tested with the CCK-8 assay. **(F)** Rabbit urethral epithelial cells were implanted with MeHA hydrogel and living cells were stained at 24 and 48 h.

In order to better simulate the natural urethra of rabbits, we extracted urethral epithelial cells and urethral smooth muscle cells from the same group of rabbits for the construction of scaffolds. And the primary rabbit urethral epithelial cells and rabbit urethral smooth muscle cells were identified by immunofluorescence staining. As shown in Figure 3A, most cells expressed CK-19, which is characteristic of epithelial cells. In addition, Figure 3B shows that most cells expressed α-SMA, which is characteristic of smooth muscle cells. The primary cells of rabbits were successfully extracted and used in follow-up experiments.

### 3.4 Biocompatibility of the PLGA/CoI electrospun membrane and the MeHA hydrogel

The ability of cells to grow and proliferate in transplants is an important factor affecting the effectiveness of urethral tissue repair. Therefore, we conducted biocompatibility experiments on PLGA/CoI electrospun membrane and MeHA hydrogel *in vitro*. The PLGA/CoI electrospun membrane was only in contact with rabbit urethral smooth muscle cells; therefore, these cells were used to test the biocompatibility of the electrospun membrane. As shown in Figure 3C, at 24 h, the survival rate of the cells implanted in the electrospun membrane did not exceed that of the control group, which is likely because the cells did not adapt to the new growth environment. At 48 h, the cell survival rate on the electrospun membrane was close to the control group. At 120 h, the survival rate had significantly surpassed that of the control group. To summarize, the PLGA/CoI electrospun membrane showed good biocompatibility with rabbit urethral smooth muscle cells and could support cell adhesion, growth, and proliferation.

The biocompatibility test of the MeHA hydrogel with rabbit urethral epithelial cells is shown in Figure 3D, and the biocompatibility test of the MeHA hydrogel with rabbit urethral smooth muscle cells is shown in Figure 3E. After 120 h of culture, when the maximum concentration of MeHA was 1%, the higher the concentration, the better the cell growth. Therefore, MeHA can obviously promote the growth and reproduction of cells. We also stained the rabbit urethral epithelial cells implanted in the MeHA hydrogel at 24 h and 48 h. As shown in Figure 3F, after 24 h culture, the cells were spherical because they were growing in the three-dimensional environment of hydrogel; the survival rate was calculated to be >99% in the live/dead cell staining. After 48 h culture, most of the cells were still spherical, but the cell density had increased greatly; the cell survival rate was still >99%, which indicated that the MeHA hydrogel had good biocompatibility with the cells, which could survive and proliferate in the MeHA hydrogel.

### 3.5 Evaluation of the effect of urethral tissue repair *in vivo*

**FIGURE 4:**
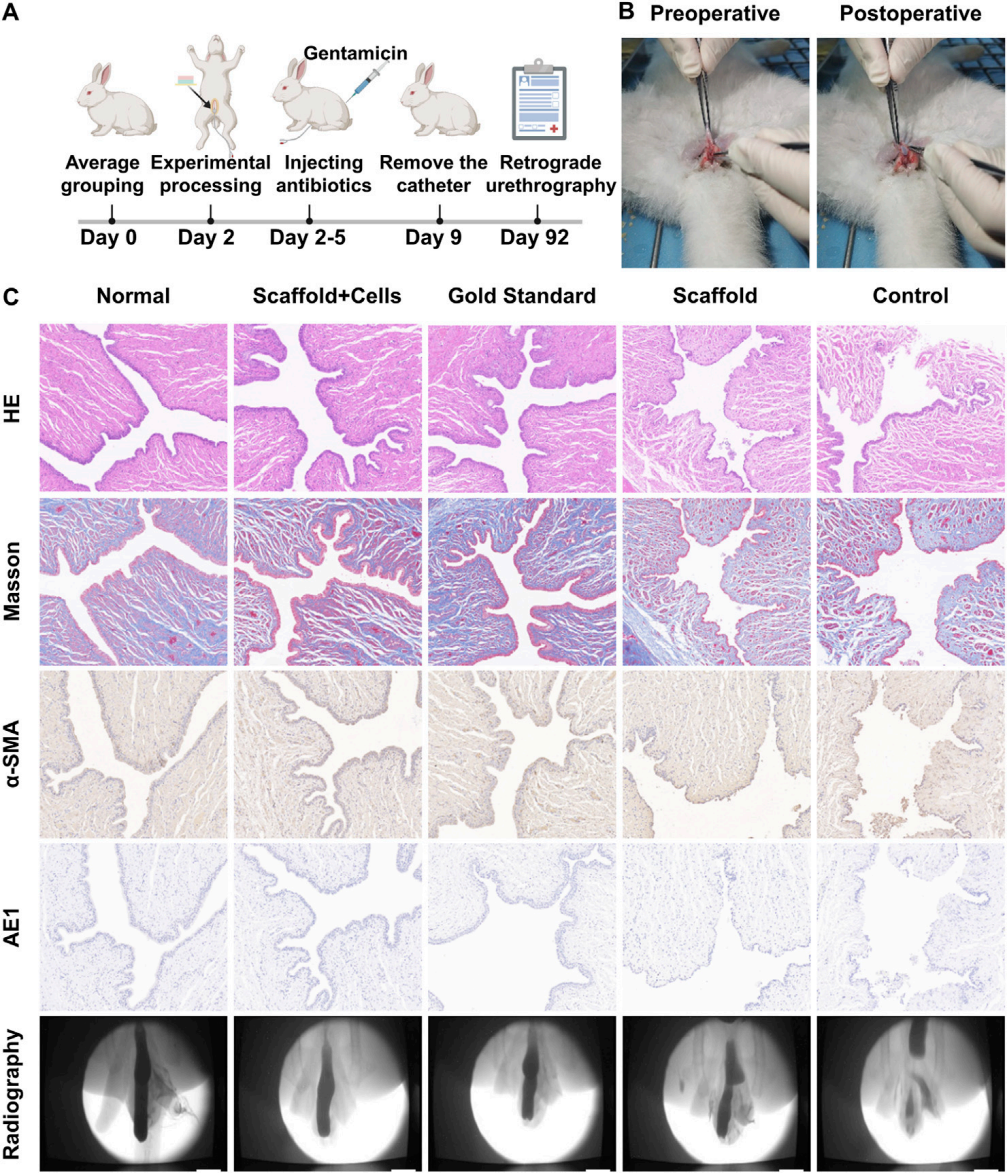
*In vivo* repair effect of the double-layer polylactic-co-glycolic acid (PLGA)/collagen type I (CoI)-methacrylated hyaluronic acid (MeHA) urethral tissue engineering scaffold. **(A)** Schematic diagram of the experimental flow chart for repairing urethral defects in rabbits. **(B)** Comparison of pictures before and after stent implantation in rabbits with the urethral defect. **(C)** Three months later, retrograde urethrography was performed in rabbit urethral defect models, and the sections were stained with hematoxylin and eosin (HE), Masson’s, anti-α-SMA, and anti-AE1.

To further validate the repair effect of PLGA/CoI-MeHA tissue engineering scaffold, we implanted the scaffold into rabbits and verified its urethral reconstruction effect 3 months later. The study rabbits were divided into four groups: PLGA/CoI-MeHA urethral scaffold implanted with cells, PLGA/CoI-MeHA urethral scaffold without implanted cells, autologous tissue suture after urethrotomy (gold standard group), and no treatment after urethrotomy (blank group). The urethral defect was established by fully separating the anterior urethra of the rabbits. Figure 4B shows the photos before and after the implantation of the urethral scaffolds. After the operation, a 6Fr catheter was inserted into the urethra and gentamicin was injected continuously for 3 days. The catheter was removed 7 days after the operation. Three months later, retrograde urethrography was performed and the repaired urethra was taken for histopathological characterization and compared with the normal New Zealand white rabbit urethra (Figure 4A). Finally, among the 40 male New Zealand white rabbits who participated in the experiment, except for the gold standard group, one rabbit died in each group; the cause of death was deemed to be infection.

The retrograde urethrography of the normal rabbit urethra and the different urethral defect models after 3 months is shown in Figure 4C. It can be clearly seen that the PLGA/CoI-MeHA urethral scaffold group with cells and the gold standard group had the best therapeutic effect; the urethral repair effect was close to that of the natural urethra. Partial stricture occurred in the urethral repair site of the PLGA/CoI-MeHA urethral scaffold group without cells, while obvious stricture or fistula occurred in the untreated blank control group. As shown in Figure 4C, the hematoxylin and eosin (HE) staining results show that the urethral epithelial cells in the PLGA/CoI-MeHA with cells treatment group and the gold standard treatment group are arranged continuously and regularly, which is very similar to the normal urethra. On the other hand, in the simple scaffold treatment group, some epithelial cells were interrupted, and the arrangement of the cells was chaotic. In the blank control group, not only were the cells disordered, an unclosed urethral fistula also appeared. Masson’s staining makes muscle fibers red and collagen fibers blue. The staining results of PLGA/CoI-MeHA with cells group and gold standard group were similar to those of the normal urethra, and the collagen fibers and muscle fibers were intertwined and evenly distributed. The scaffold treatment group and the blank control group showed different degrees of collagen fiber agglomeration, suggesting that there may have been a fibrous scar causing urethral stricture or obstruction. α-SMA and AE1 were used to label smooth muscle cells and epithelial cells, respectively, which were consistent with the results of the above sections. The therapeutic effect observed in the PLGA/CoI-MeHA with cells treatment group and gold standard treatment group was close to that of the normal urethra, and the arrangement of smooth muscle cells and urethral epithelial cells was regular. However, the arrangement of cells in the scaffold treatment group and blank control group was complicated, which was significantly different from that of normal urethra.

In summary, PLGA/COI-MeHA tissue engineering scaffold has a significant effect on urethral repair *in vivo*. The reconstructed rabbit urethra is close to the natural urethra in terms of imaging representation and slice data, making it a very promising urethral repair graft.

## 4 Discussion

Our research illustrates that the double-layer PLGA/COI MeHA scaffold for tissue engineering effectively facilitates urethral reconstruction. Utilizing hydrogel, the arrangement of urethral cells is significantly enhanced. Both electrospinning and hydrogel contribute substantially to support and stabilization.

In treating urethral repair, some researchers now employ engineered nanofiber scaffolds to efficiently promote the development of nearby blood vessels, achieving beneficial outcomes (Niu et al., 2020; Wan et al., 2020). Additionally, the engineered urethral scaffold helps stimulate the proliferation of epithelial and smooth muscle cells, thereby supporting urethral repair (Liu et al., 2020). Its interaction with stem cells represents another promising avenue for advancement (Zhu et al., 2022). Unlike oral mucosal grafts (Sánchez et al., 2023b), tissue engineering scaffolds eliminate damage to the donor site and are generally easier to procure. Their favorable tissue compatibility and effectiveness in urethral reconstruction are crucial for clinical implementation. However, current techniques for urethral restoration primarily involve surgically implanting grafts, which unavoidably inflicts a certain degree of trauma on patients. Consequently, the challenge lies in refining treatment techniques to minimize both complexity and injury while maintaining effective therapeutic outcomes.

## 5 Conclusion

Through electrospinning and light curing technology, we successfully screened the PLGA/CoI (7:3) electrospun membrane and MeHA (40.72%) hydrogel. Furthermore, we successfully prepared PLGA/CoI-MeHA bilayer urethral scaffolds loaded with rabbit urethral smooth muscle cells and rabbit urethral epithelial cells, respectively, and achieved favorable results for urethral defect repair and urethral reconstruction in rabbits. The mechanical characterization of the scaffold indicates that it has sufficient mechanical strength to meet experimental and clinical needs. In addition, it showed satisfactory biocompatibility in cell experiments and in the *in vitro* degradation experiments. The excellent performance of the PLGA/CoI-MeHA double-layer urethral scaffold in repairing urethral defects in rabbits suggests that it may be effective for clinical repair of the human urethra.

## Data Availability

The original contributions presented in the study are included in the article, further inquiries can be directed to the corresponding author.
